# He, She, “They” at the Bargaining Table… Woman, Man or Just Negotiators? A Critical Review on Gender Ideologies in Mixed-Gender Negotiations

**DOI:** 10.5334/pb.523

**Published:** 2020-07-28

**Authors:** Claudia-Neptina Manea, Vincent Yzerbyt, Stéphanie Demoulin

**Affiliations:** 1 Faculty of Psychology and Educational Sciences, Université catholique de Louvain, BE

**Keywords:** gender, gender ideology, negotiation, gender differences

## Abstract

In recent years, an important number of studies aimed at levelling the playing field for women and men at the bargaining table. In spite of this, women continue to find themselves largely at a disadvantage when negotiating with a male counterpart. In this review, we focus on a neglected yet potentially important factor contributing to the gender gap in negotiations, namely people’s gender ideology. We argue that gender ideology shapes day-to-day negotiations and we offer insights regarding its consequences on the bargaining process. We first present the available contributions regarding gender in negotiations and underline the most significant variables that lead to gender effects in negotiations. We then introduce gender ideology as a potentially important variable in the process and lean upon previous studies to support our claim.

Imagine that you are about to start a negotiation with a partner of the opposite sex. Does the fact that you are a woman and that he is a man, or the other way around, play a role in the choices that you are about to make when striving to reach your goals? Will you approach the negotiation the same way that you would if your counterpart were of the same gender^1^ as you? Decades of research on gender in negotiation argue that women negotiators suffer a disadvantage on a number of counts. Many of the mechanisms leading to these gender effects have already been underlined and scholars have now started investigating the various ways by which they could be overcome (e.g., Kray, Galinsky, & Thompson, 2002; Small, Gelfand, Babcock, & Gettman, 2007). In the present paper, we expand this literature by examining how the negotiation process could be influenced by gender ideologies, i.e., people’s beliefs of whether and how gender ought to be either ignored or acknowledged for reaching equality (Koenig & Richeson, 2010).

We start by presenting research that portrays gender as an important determinant of outcomes at the bargaining table. We first point to the variables used to account for women’s poorer performances in negotiations. In particular, we approach individual differences in female and male negotiator’s management of the mixed-gender bargaining table. We then turn to the partners’ differential reactions towards women and men as negotiators partners. We complete this analysis by considering situational factors operating independently of negotiators and their partners’ behaviors. We then redirect our attention to a heretofore neglected yet potentially important factor to increase our understanding of gender effects in negotiation, namely the various beliefs that people hold as to how gender is and ought to be handled in daily-life interactions, i.e., gender ideologies. We present the state of the art on these gender beliefs. We then build upon this literature to argue that one way of handling one’s gender in negotiations is to vary its salience as a function of both the congruence of the task with the negotiator’s gender (congruent vs. incongruent task) and the expected outcomes of the bargaining process (wanted, positive vs. unwanted, negative outcome). We then exemplify how this model applies in the case of women negotiators before concluding that gender ideologies constitute a valuable research topic for future studies on gender effects in negotiation.

## Gender Effects in Negotiation

Abundant research findings have shown that women’s performance in mixed-gender negotiations often fall below those of men, especially in negotiations on monetary stakes (e.g., Bowles, Babcock, & McGinn, 2005; Stevens, Bavetta, & Gist, 1993; Stuhlmacher & Walters, 1999; Walters, Stuhlmacher, & Meyer, 1998). Over the years, scholars have conducted a host of studies to uncover the mechanisms that may account for this gender gap. Broadly speaking, three kinds of variables have been found to account for these gender effects: individual differences between female and male negotiators, partners’ differential reactions to women and men negotiators, and situational factors (for a review, see Demoulin, 2014). The following section assesses how each of these factors affects women’s negotiating behaviors and performances.

### Gender Differences among Negotiators

Research points to behavioral differences between female and male negotiators in all phases of the negotiating process, that is, before, during, and after the negotiation per se. *Before the negotiation*, women are less likely to perceive a given situation as being negotiable than men are (Babcock, Gelfand, Small, & Stayn, 2006). This leads them to avoid the bargaining table altogether, particularly when the likelihood of negative consequences is high (Exley, Niederle, & Vesterlund, 2016). When negotiation is unavoidable, women also set lower goals and have lower expectations than their male counterparts do (Kray, Thompson, & Galinsky, 2001; Major & Konar, 1984; McFarlin, Frone, Major, & Konar, 1989).

*During the actual negotiation process*, female negotiators commonly speak less and show more self-doubt than male ones (Kimmel, Pruitt, Magenau, Konar-Goldband, & Carnevale, 1980). They also react in a more emotional manner (Stuhlmacher & Walters, 1999) and tend to consider what happens as part of a long-time relationship whereas men usually portray each negotiation episode as a separate, unconnected event (Greenhalgh & Gilkey, 1993). Consequently, women often show more interest in interpersonal relationships at the bargaining table (Kray & Gelfand, 2009), and are more willing to share personal information than men (Halpern & McLean Parks, 1996). In other words, they end up significantly more on the cooperative side than their male counterparts (Anderson & Shirako, 2008) who are instead more likely to endorse a more competitive perspective (Walters et al., 1998).

Gender differences also affect ethical behaviors in negotiation. Women use positional commitments, threats, and derogatory put-downs to a lesser extent than men do (Kimmel et al., 1980; Lewicki & Robinson, 1998; Robinson, Lewicki, & Donahue, 2000). They are also less egocentric in negotiation ethics than men (Kray & Haselhuhn, 2012) and feel less comfortable with higher financial compensation (Barron, 2003). On a similar note, when participating in allocation tasks, women pay themselves less than male participants do (Kaman & Hartel, 1994; Major, McFarlin, & Gagnon, 1984) and sometimes even less than other people are willing to pay them (Callahan-Levy & Messe, 1979).

*At the end of a negotiation episode*, women report less satisfaction with their overall performance than men do (Watson & Hoffman, 1996). They acknowledge feeling less powerful during the bargaining process (Kray et al., 2001) and report greater dislike of the whole process (Babcock et al., 2006; Kimmel et al., 1980; Small et al., 2007) as well as lower self-efficacy (Stevens et al., 1993).

### Differential Reactions among Negotiation Partners

As such, the above-mentioned findings might lead one to consider that empowering women would suffice to erase gender effects in negotiation. However, negotiations do not take place in a social vacuum (Kray & Gelfand, 2009). A woman’s ability and motivation may thus not be the only aspects that influence the outcomes. Rather, her counterpart’s approach also plays an important role in the way negotiations evolve. In particular, several studies reveal that partners treat men and women differently in negotiations, even when the latter negotiate identically. For instance, research has shown negotiators to be four times more likely to deceive a female than a male counterpart (Kray, Kennedy, & Van Zant, 2014). Because one expects women to be warm and kind, they often hesitate to accuse others of foul play, as such accusations would violate prescriptive feminine stereotypes (Kray, 2011). Unfortunately, this double bind also opens the door to the prevalent stereotype of gullible women, and implicitly to even stronger attempts at deceiving female negotiators (along similar lines, see Smeesters, Warlop, Van Avermaet, Corneille, & Yzerbyt, 2003).

Similarly, men are often better rewarded for their work and are free to succeed without being disliked or sabotaged (Correll, Benard, & Paik, 2007; Kilbourne, England, Farkas, Beron, & Weir, 1994). Men also often receive better offers in negotiation (e.g., Ayres & Siegelman, 1995) and, thus, as a consequence of an anchoring effect, obtain better results at the end of the discussion. Meanwhile, women’s success emerges as much more likely to be accompanied by various negative consequences (Amanatullah & Tinsley, 2013b; Bowles, Babcock, & Lai, 2007; Rudman & Fairchild, 2004; Rudman & Phelan, 2008). For instance, research has shown that women are reprehended more than men for negotiating on their own behalf (Amanatullah & Tinsley, 2013a; Bowles et al., 2007). That is, the prevalent stereotype that assigns communal traits to women (Williams & Best, 1982) and agentic characteristics to men creates a double bind and exposes women to social backlash when focusing on personal goals (Amanatullah & Morris, 2010).

### Situational Factors

Recent research on gender effects in negotiation has also envisaged the situational factors that exacerbate versus reduce gender differences. Most studies in this domain argue that gender gaps result in part from the differential roles ascribed to women and men in the society and from the stereotypical traits associated to these roles (Eagly & Steffen, 1984). Because people consider negotiation as a male prerogative (Bowles & Kray, 2013) and because performance in negotiation is associated with stereotypical masculine characteristics (Kray et al., 2001; Williams & Best, 1982), negotiations are threatening to most women (e.g. Small et al., 2007). Indeed, studies reveal, for instance, that women tend to avoid the bargaining process when situations are framed as opportunities for negotiation. However, simply reframing negotiations as opportunities to ask is apparently enough to eliminate this gender gap in the initiation of negotiation (Small et al., 2007). On a similar note, women respond better to the challenge when they envision the upcoming negotiation as a learning tool, as opposed to when they believe it to be diagnostic of their true ability (Kray et al., 2001). Finally, women’s performance also significantly increases when negotiating for someone else as opposed to negotiating for themselves (Bowles et al., 2005). According to the authors, the latter effect occurs because the representational role is in line with women’s communal stereotype and caring role in general.

In their meta-analysis on gender effects, Stuhlmacher and Walters (1999) further point out that most of the studies that evidenced a male advantage on negotiations use stereotypically masculine negotiation tasks as well as highly competitive contexts. Indeed, recent research confirmed that when feminine topics are at stake or when contexts are cooperative, women are just as willing and capable to successfully negotiate as their male counterparts (Babcock, 2014; Bear, 2011; Bear & Babcock, 2012). In fact, many studies have now acknowledged that, under appropriate circumstances or adequate framing, the gender gap can be erased (e.g., Kray & Gelfand, 2009; Leibbrand & List, 2015; Small et al., 2007). Such studies can only point to the importance of keeping an open mind when investigating the influence of gender in mixed-gender negotiations. As past research suggests, women might indeed underperform relative to men in negotiations, but only under specific circumstances. Critically, factors other than a presumed woman’s deficiency at the bargaining table appear to account for a great deal of the discrepancies in mixed-gender negotiations. A clearer insight on these variables and on their effects on women’s negotiating performances is obviously of utmost importance.

## Gender Ideologies

Although past research has undeniably improved our understanding of the emergence of gender differences at the negotiation table, we are yet to fully understand gender effects in mixed-gender negotiations. Little is known, for instance, on the role that people’s beliefs regarding how gender is and should be approached at the negotiation table, namely their gender ideologies, play on the matter. Hereafter, we argue that gender ideologies are important in determining how mixed-gender negotiations unfold. As such, we first point to past work focused upon one’s ideological endorsement on daily mixed-gender interactions. We then build upon this literature to argue that gender ideology is potentially relevant to better understanding gender effects in negotiations.

### Theoretical Models on Gender Ideologies

For years, the topic of how gender should be handled when women and men interact has escaped the interest of social psychologists. Recently however, two models emerged to shed light on the possible beliefs people hold on gender differences in mixed-gender interactions. The first model suggests that one can either acknowledge or ignore gender in mixed interactions (Koenig & Richeson, 2010). The second model additionally distinguishes between positive and negative ways to acknowledge or ignore gender differences (Hahn, Banchefsky, Park, & Judd, 2015).

Building upon research that deals with racial ideologies and in which researchers make a distinction between a colorblind and a multicultural approach to racial encounters (e.g., Richeson & Nussbaum, 2004; Wolsko, Park, Judd, & Wittenbrink, 2000), Koenig and Richeson (2010) proposed to consider two possible ways of handling gender differences in daily interactions. On the one hand, the sexblind ideology holds that, in order to increase gender equality, one should eliminate the use of sex categories and approach both men and women as unique individuals. On the other hand, to attain the same goal, the sexaware ideology maintains that one should acknowledge and celebrate sex differences instead. In other words, this model envisions sexblindness and sexawareness as opposite ends of a continuum and uses items such as “There is no reason to categorize individuals as men or women” (sexblindness) or “Both men and women have unique assets to contribute” (sexawareness) to identify one’s tendency to acknowledge/ignore gender.

More recently, Hahn et al. (2015) enriched Koenig and Richeson’s proposition by crossing the importance granted to gender differences with the positive or negative evaluations of subordinate group members. As a result, this model produces a *fourfold framework* of gender ideologies. According to the sexblind^2^ perspective, the best way to handle gender is to ignore the category altogether and to value individuals (e.g., “All humans are fundamentally the same, regardless of their gender”). In contrast, assimilationism proposes a negative evaluation of the subordinate group and states that gender equality can best be achieved by assimilating one gender to the norms imposed by the other. Specifically, one expects women to assimilate to men’s norms (e.g., “Women in the corporate world should embrace a masculine work ethic”). Turning to ideologies that celebrate gender differences, the sexaware perspective emphasizes differences between women and men and promotes the idea that both genders hold equally important qualities, albeit different ones. As such, gender differences come across as mutual enrichment (e.g., “Men and women have different but equally useful ways of accomplishing tasks”). In contrast, when the evaluation of the subordinate group is negative, i.e., segregationism, the differences between women and men are used to keep the genders apart and to justify, at least at an implicit level, placing women in a lower status and in less desirable positions than men (e.g., “Men and women are naturally suited to different jobs and should continue to do those”).

Importantly, the relevance of these gender ideologies should vary as a function of context. For instance, not all ideologies should apply to the field of interest of this article, i.e., the particular domain of *negotiations*. As Hahn and colleagues (2015) argue, segregationism advocates the fact that women and men should be kept apart. However, working together towards a mutually acceptable solution is a key aspect of the bargaining process. In other words, interpersonal interaction is not a matter of choice but a required condition. Considering this, sexblindness, sexawareness, and assimilationism should be the ideologies most likely to actively emerge within mixed-gender negotiations.

### Antecedents of Ideologies’ Endorsement

People’s way of approaching gender differences varies along with the concrete circumstances that they encounter as well as with whom they are. Indeed, several factors, both situational (e.g., context, the diversity norms within a particular organization, one’s cultural background or their prototypicality to the considered field) and individual (e.g., one’s gender, level of prejudice, or personal values), were recognized over the last decade to interact in determining people’s endorsed gender ideologies.

Preliminary studies conducted by Koenig and Richeson (2010) suggest for instance that context influences one’s ideological endorsement. Indeed, whereas people embrace sexblindness more in work environments, they prefer sexawareness in social settings. Individual differences would also seem to play a role. For instance, the preference for sexblindness is related to less benevolent sexism in social interactions. Although the same pattern can be found in work contexts, the differences do not reach statistical significance (Koenig & Richeson, 2010), suggesting that context and individual differences work together in determining people’s choice of gender ideologies.

In a recent study on equality policies and attitudes in Luxemburg, Bourguignon, Tisserant, Fointiat, Grézault, Leymarie, Heiser & Wagner (2015) report new evidence on the way situational and individual variables interact in shaping people’s ideological approach in work contexts. As expected, the diversity norms promoted within an organization emerged as the best predictor of people’s endorsements of gender ideologies. However, individual factors were again found to be of importance. Although participants overall preferred positive gender ideologies (sexblindness and sexawareness) to negative ones, male participants supported sexblindness more than women did. Moreover, a high level of social dominance orientation related to a higher preference for negative gender ideologies, whereas a low level was related to a preference for positive ones. This study also revealed the impact of one’s cultural background on the endorsement of gender ideologies. More specifically, participants originating from Luxemburg supported negative gender ideologies (assimilationism and segregationism) more, and sexblindness less, than employees of other nationalities did (i.e., mostly French and Belgians, but also Italians, Portuguese, Germans, etc.). The latter result does not come as a surprise considering Guimond, de la Sablonnière and Nugier’s (2014) findings that intergroup ideologies, institutionalized as policies, often vary as a function of culture and time.

More recently, Banchefsky and Park (2018) further advanced our understanding by looking at the match between gender imbalance within a given study major and women and men’s endorsed gender ideologies. Again, situational (i.e., the gender balance within the study major) and individual factors (i.e., gender) interacted in determining people’s endorsement of gender ideologies. More specifically, gender imbalance in the study major was not found to affect women’s gender ideologies. In contrast, men in increasingly male-dominated academic majors proved more likely to endorse negative gender ideologies (assimilationism and segregationism) and less likely to endorse sexblindness. The data revealed no impact of the relative percentage of men and women in the study major on their support for sexawareness (Banchefsky & Park, 2018).

These are obviously not the only individual factors to affect people’s gender ideologies. In a recent study, for instance, Martin, Gündemir, Phillips and Homan (2018) found women’s values to also be of importance when it comes to the gender ideologies they endorse. As research shows, women holding strong career values (i.e., those who prioritize career-related goals) prefer sexblindness. In contrast, women with stronger family values (i.e., those who prioritize family-related goals) support sexawareness more.

In sum, the available work portrays gender ideology as a promising concept but suggests that various situational variables (including, but not limited to context, cultural norms and one’s prototypicality to a particular field) seem to interact with individual ones (e.g., gender and personal values) in orienting the preferred gender ideology.

## Gender Ideologies in Negotiation

Gender ideology represents a rather abstract concept, one that obviously needs to translate into concrete actions (i.e., gender ideology-based strategies) to impact mixed-gender negotiations. Moreover, with one exception (Manea, Demoulin, & Yzerbyt, 2019), previous research has not examined the impact of gender ideologies on negotiations. In the present section, we take a step forward as we explore the concrete ways in which gender ideologies should manifest themselves at the negotiation table. More specifically, we propose a theoretical model that suggests that people’s strategic preferences for putting forward their gender or for attempting to downplay it vary as a function of the task’s congruence with their gender (i.e., congruent vs. incongruent task) as well as of the expected outcome of the negotiation at hand (i.e., wanted, positive vs. unwanted, negative outcomes). That is, we posit that gender congruence with the task is likely to be perceived as beneficial to individuals when negotiating in order to obtain a wanted, positive outcome. The same gender congruence might however be seen as decreasing their chances of success when the goal is to avoid unwanted, negative outcomes. In a similar vein, a lack of gender congruence with the task should be perceived as a liability when aiming to obtain a positive, wanted outcome in mixed-gender negotiations whereas the same lack of gender congruence should instead be seen as an advantage when negotiating over an unwanted task.

Clearly, this model should apply equally to female and male negotiators. Indeed, as role congruence theory (Eagly, 1987) posits, both women and men feel more at ease in gender congruent contexts and prefer gender congruent to gender incongruent tasks. Moreover, the literature on ambivalent sexism found gender to represent only a minor predictor of sexist attitudes (Roets, Van Hiel, & Dhont, 2012). Although gender ideologies and sexism are obviously different concepts, one can nevertheless hardly ignore the overall similarity in women’s and men’s perspectives pertaining to both the importance of gender congruence, and to how women and men should overall behave.

As much as this, we will detail the proposed framework by focusing primarily on *women*. Indeed, as already argued and as detailed in the first section of the present work, negotiation is not only considered mostly as a masculine activity that takes place at work but negotiation research has also focused mainly on women’s deficiencies at the bargaining table (Kray & Thompson, 2005; Williams & Best, 1982). Better understanding how women handle (their) gender in this context thus seems particularly important, as it should offer additional insight on previously found gender effects in negotiation.

### Handling Gender in Mixed-Gender Negotiations

Gender equality is an important matter on today’s societal agenda (*Strategic engagement for gender equality 2016–2019*). More than ever, people are reminded of the importance of egalitarian and undifferentiated treatments of both genders. In most cases, this attempt to ensure equal chances for women and men translates into norms that prescribe gender to be ignored and all decisions be made solely based on people’s individual abilities (i.e., sexblindness). This posture is particularly salient in professional environments (Koenig & Richeson, 2010) and this is bound to affect the mixed-gender bargaining table. Given the high stakes generally associated to negotiations, women (just like men) should overall be even more careful not to deviate from the prescribed behavior (i.e., sexblindness). As such, gender downplaying should represent the overall baseline in situations of negotiation, particularly within professional contexts.

Whereas sexblindness should indeed represent the golden rule at the bargaining table, this perspective might not be the only one people rely upon in mixed-gender negotiations. As we will show below, depending on the joint effect of task’s gender congruence (congruent vs. incongruent) and of the expected (wanted vs. unwanted) negotiation outcome, both sexawareness and assimilationism might, at times, be considered as valid options for female (and male) negotiators.

#### Handling Gender in Gender-Incongruent Contexts (i.e., Women in Male-Congruent Negotiations)

Lay perceptions consider masculine competitive work contexts as the golden standard in negotiations. It should therefore not come as a surprise that most studies to date focused upon organizational environments, masculine tasks, and highly competitive negotiations (Stuhlmacher & Walters, 1999). Women and men’s abilities are thus typically investigated under rather specific conditions, where men’s higher gender-role congruence grants them a privileged position (along the same lines, see Wood & Karten, 1986; Wood & Rhodes, 1992). In other words, the stereotype of a good negotiator is not far from the one of men (Kray et al., 2001), and negotiations are mostly considered as more of a male domain (Raiffa, 1982; Williams & Best, 1982). Clearly, as role congruity theory (Eagly & Karau, 2002) suggests, the lack-of-fit women experience in such negotiations is bound to negatively affect their performance. Indeed, given this male stereotypical – dominance in negotiations (Kray & Thompson, 2005), women should have no interest in commonly acknowledging (their) gender in this domain. Because women negotiators typically emerge as both less willing and less able to negotiate on their behalf (e.g., Babcock & Laschever, 2003; Babcock et al., 2006; Kimmel et al., 1980; Stevens et al., 1993), it seems reasonable to assume that they would perceive their gender as a liability at the typical bargaining table. This reasoning is consistent with past research on the “queen bee” phenomenon. According to this line of work, women who pursue individual success in male-dominated settings often adjust to the dominant, i.e., masculine, culture and end up distancing themselves from other women (see, for instance, Derks, Van Laar, & Ellemers, 2016). Similarly, in male-dominated negotiation contexts, women aiming to obtain positive (i.e., wanted) outcomes (e.g., a salary raise) should likely prefer assimilationism over sexawareness.

At the same time, although women should commonly opt for downplaying gender in male-congruent negotiations, the expected outcome should also influence this choice. Specifically, sexawareness might be seen as a more effective manner to handle the discussion when trying to avoid negative (unwanted) outcomes (rather than trying to obtain positive, wanted ones). That is, when trying to avoid a traditionally male-congruent activity (e.g., mowing the lawn), acknowledging gender might offer women an (welcomed) advantage, by reminding their male counterparts about women’s lack-of-fit in such tasks. In other words, when trying to avoid unwanted gender-incongruent matters, sexawareness may prevail over assimilationism.

#### Handling Gender in Gender-Congruent Contexts (i.e., Women in Female-Congruent Negotiations)

Although lay beliefs often associate mixed-gender negotiations to stereotypically male bargaining contexts, this is not always the case. Indeed, negotiations are a ubiquitous part of daily life and significantly affect all aspects of one’s existence. Interestingly, under certain circumstances (e.g., when negotiating on a feminine topic, or on behalf of others), the masculine ‘tone’ of the bargaining table is reduced, and women’s perceived incongruence seems to be (at least partly) minimized. Moreover, in such female-congruent contexts, gender effects shrink or vanish entirely (for compelling examples, see Amanatullah & Morris, 2010; Bear, 2011; Bear & Babcock, 2012; Wade, 2001). Indeed, feminine contexts are known to benefit women negotiators. Female-congruent discussion topics, for instance, seem to positively affect their performances, by making them both more willing, and more able to negotiate for a result in their favor (Bear, 2011; Bear & Babcock, 2012; Miles & LaSalle, 2008). As the saying goes, “knowledge is power”, and the feeling of power significantly reduces gender differences in both first offers and negotiating outcomes (Hong & van der Wijst, 2013). Other variables, such as the representation role, more congruent to women’ s prescribed caring and other-oriented nature (Eagly, 1987), have equally been shown to diminish women’s fear of negative repercussions, thus enhancing their overall negotiating performances (Amanatullah & Morris, 2010; Bowles et al., 2005; Wade, 2001).

In line with this literature, one could speculate that women’s strategical choice to gender acknowledgement in female-congruent negotiations would differ from their preferences in male-congruent situations. We propose that, in such circumstances, women should be prone to capitalize upon their (undue) gender leverage in more female-congruent circumstances than they are in male-congruent ones. Negotiations concerning various family and childcare matters (traditionally considered as a woman’s prerogative), for instance, should have female negotiators more readily acknowledge gender. Because women presumably hold more expertise in these domains, they should use this leverage to impose their perspective. Said otherwise, women should endorse sexawareness over assimilationism when trying to obtain a positive, wanted outcome on an issue that is congruent with their gender.

Then again, although most feminine contexts would seem to favor women, by making them both more willing and more able to negotiate (Bear, 2011; Bear & Babcock, 2012), a higher gender congruence may not always benefit them. Indeed, this increased gender congruence may represent a liability for female negotiators when trying to avoid duties and other unpleasant tasks stereotypically assigned to women (e.g., various household chores, Organization for Economic Cooperation and Development [OECD], 2019). Said otherwise, along with the possible benefits stemming from women’s greater gender congruence with the negotiation topic (Bear, 2011), gender congruence can also lead to negative consequences when the topic of discussion focuses on unwanted, negative outcomes (*see* Demoulin & Teixeira, 2016), by activating the stereotype that some tasks represent “a women’s job”. In such contexts, sexawareness may end up legitimizing gender roles, building on the idea that women are better fitted to handle these tasks than men. In these cases, women may be less tempted to acknowledge (their) gender and instead downplay gender. In other words, women may prefer assimilationism over sexawareness when trying to avoid an unwanted outcome on a task that is congruent with their gender.

### Consequences of Different Gender Ideologies in Mixed-Gender Negotiations

Given the above mentioned literature on gender ideologies and gender in negotiation, both gender congruence with the task (i.e., gender congruent vs. gender incongruent task) and expected outcomes (i.e., wanted vs. unwanted outcomes) would indeed seem to influence how one handles gender during the bargaining process.

Then again, the choice to either acknowledge or ignore gender in mixed-gender negotiations should hardly remain without consequences. For this reason, a better understanding of the potential impact of gender ideologies in this context is obviously important.

#### Consequences of Gender Ideologies in Standard (i.e., Male-Congruent) Negotiations

Building upon literature, women’s presumed first choice of gender ideology in negotiation, i.e., sexblindness, should indeed represent their best possible option with respect to gender handling in standard negotiation contexts. More specifically, by redirecting attention from one’s gender to their individual traits, sexblindness is likely to reduce women’s lack-of-fit in negotiations. As past research reveals, when it comes to work contexts, sexblindness increases women’s confidence and actions necessary for reducing gender disparities (Martin & Phillips, 2017; Torres & Martin, 2018). This means that sexblindness emerges as a more effective approach for women in such situations (Gündemir, Martin, & Homan, 2019). In other words, downplaying gender at the bargaining table by focusing on other (non-gender related) traits should benefit women more than focusing upon gender differences (i.e., sexawareness) or trying to behave more like a man (i.e., assimilationism).

Interestingly enough, research on racial ideologies sends a cautionary note with respect to this general prediction. Indeed, several efforts suggest that downplaying differences is not always the answer and that acknowledging the differences is often more beneficial to minorities (see, for instance, Plaut, Thomas, & Goren, 2009). This means that, next to the obvious benefit of redirecting attention towards other (non-gender related) traits, sexblindness may also entail the disadvantage of downplaying long existing stereotypes that hardly disappear just because one chooses to look away. Clearly, future research should explore this matter further.

Turning to assimilationism, the literature on the backlash effect (Bowles et al., 2007; Rudman, 1998; Rudman & Glick, 1999, 2001; Stuhlmacher & Linnabery, 2013) would lead us to question the benefits of this strategy when women try to obtain a positive, wanted outcome in gender-incongruent negotiations. Although people believe to some extent that behaving like a man offers a clear path to success (Manea et al., 2019), research shows that women who behave in a gender-incongruent manner question their prescribed role and implicitly risk negative reactions. Bowles and colleagues (2007), for instance, found male evaluators to penalize women more than men for attempting to negotiate for higher compensation (i.e., a stereotypically male behavior). More specifically, when choosing potential coworkers, men preferred nicer, less demanding women who readily accepted their compensation offers to those who had attempted to negotiate, though all women appeared equally competent. As for women’s reactions, their behavior varied across the studies. Whereas in some experiments the target’s gender did not influence women’s evaluations, in other studies women (just like men) indeed penalized other women more than they did men for attempting to negotiate (Bowles et al., 2007). As such, assimilationism would most likely work against, rather than in favor of women negotiators when aiming to obtain a wanted outcome in a negotiation that is incongruent to their gender.

Turning to sexawareness, past work offers mixed conclusions as to whether this strategy represents a viable option for women negotiators. At first glance, given the male-domain connotation of negotiations (Raiffa, 1982), acknowledging gender would seem to increase women’s awareness on their presumed incongruence at the bargaining table, thus negatively impacting their self-confidence – and implicitly their overall performance. The literature on feminine charm would however disagree. Kray, Locke, and Van Zant (2012), for instance, show that when women negotiators use feminine charm they are perceived as more effective, having greater understanding of their negotiating partner’s interests, and enhancing the positive mood of their interaction partner. Moreover, depending on its balance of friendliness and flirtatiousness, feminine charm has the potential to influence the way partners divide resources in mixed-gender negotiations (Kray et al., 2012). Though not explicitly focused on gender ideologies, studies such as these suggest that acknowledging the added value of gender differences might benefit rather than hurt women at the bargaining table, even when trying to obtain a benefit on tasks that are not congruent with their gender.

Importantly, women’s choice to acknowledge gender should be particularly profitable when bargaining to avoid tasks that are not congruent to their gender. In such cases, by making gender salient, women may indeed manage to avoid male-congruent duties (i.e., use gender stereotypes to their benefit). Future research should definitely assess the role of gender ideologies within mixed-gender negotiations on tasks that are gender-incongruent.

#### Is Sexawareness the Answer for Women in Female-congruent Negotiations?

As our model posits, gender congruent contexts are significantly more likely than gender incongruent ones to elicit gender acknowledgement when positive outcomes are expected. Indeed, increased sexawareness may (at least partly) explain the positive effect of feminine contexts on women’s negotiating outcomes when trying to obtain a positive, wanted outcome (e.g., Amanatullah & Morris, 2010; Bear, 2011; Bear & Babcock, 2012; Bowles et al., 2005; Miles & LaSalle, 2008). That is, although downplaying gender may constitute a more effective way of handling male-based negotiations than acknowledging gender, women should nevertheless benefit from perceiving their gender as an asset rather than as a liability. Past work on other diversity ideologies argues in favor of acknowledging (rather than downplaying) interindividual differences. That is, multiculturalism (the equivalent of sexawareness’s in the domain of racial ideologies) was found to positively influence interracial interaction, engagement, performance, and detection of discrimination (for further information, see Plaut et al., 2009). By increasing women’s confidence in their capacity to succeed, this change in perspective should prove equally beneficial to women, leading toward better performances in gender-congruent negotiations as compared to assimilationism or even sexblindness.

Then again, as already mentioned, the same sexawareness that likely benefits women when aiming for a positive, wanted outcome in gender-congruent negotiations, might equally explain their poorer performance when bargaining to avoid an unwanted outcome on tasks that are congruent to their gender (Demoulin & Teixeira, 2016). Indeed, whereas women should find sexawareness beneficial when trying to obtain a positive, wanted outcome in contexts that are gender-congruent, things may be quite different when trying to avoid a task that is stereotypically assigned to their gender. In such cases, sexawareness may likely hurt rather than help women’s chances of success in negotiations. In other words, when negative outcomes are expected in female-congruent negotiations, women should benefit more from ignoring gender (i.e., sexblindness, or even assimilationism) than from acknowledging it (i.e., sexawareness). Future research should try to understand how and when sexawareness benefits negotiators, and how gender congruence of the task and the expected outcomes interact in determining their gender approach in negotiations.

## Future Directions of Gender Ideology Research in Negotiation

We began this paper by asking whether one’s gender beliefs might be partly responsible for the significant gender effects reported in the negotiation literature. As our analysis suggests, gender ideology seems to play an important part in how negotiations evolve. As such, better understanding when and under what circumstances gender beliefs affect mixed-gender negotiations is of the utmost importance.

To shed light on this matter, we proposed a model that posits a joint effect of the gender congruence of the task (congruent vs. incongruent task) and of the expected (wanted vs. unwanted outcome) outcomes on the way gender is approached in mixed-gender negotiations. We presented our model by focusing upon women negotiators and argued that women are more likely to acknowledge gender when trying to obtain a wanted outcome in a feminine (i.e., gender-congruent) context (e.g., jewelry negotiation, Bear & Babcock, 2012) or to avoid an unwanted outcome in a masculine (i.e., gender-incongruent) negotiation (e.g., mowing the lawn, Demoulin & Teixeira, 2016). In contrast, when trying to obtain a desired outcome in masculine negotiations (e.g., monetary negotiations, Stuhlmacher & Walters, 1999) or when trying to avoid a female-congruent task (e.g., doing the dishes, Demoulin & Teixeira, 2016), women would likely prefer to downplay their gender. Future studies should put this theoretical framework to a test, and explore, for instance, how various bargaining circumstances influence the way women handle (their) gender in negotiations. Indeed, better understanding women’s gender approach in contexts that increase (as opposed to diminish) their negotiating opportunities (e.g., feminine topics, Bear, 2011; Bear & Babcock, 2012 or representational roles, Bowles et al., 2005) should shed additional light on gender effects in negotiations and even contribute to a new perspective on gender equality. That is, if gender acknowledgement should indeed be found to level the field in some negotiations, as our model would suggest, then current societal norms positing gender downplaying as *the* golden rule for gender equality obviously need reconsideration.

From a somewhat complementary perspective, past work would argue that negotiations do not happen in a vacuum (Kray & Gelfand, 2009), and women’s way of handling gender is bound to be affected by their male counterpart’s approach. As such, better understanding men’s gender approach in negotiations would seem more than relevant. Of importance, our model posits that men’s approach should follow similar guidelines as the one previously exemplified for women. Like women, men should benefit from acknowledging gender when bargaining to obtain a wanted outcome on tasks that are congruent to their gender (e.g., monetary purchases, Stuhlmacher & Walters, 1999) or when aiming to avoid duties stereotypically assigned to women (e.g., doing the dishes, Demoulin & Teixeira, 2016). Then again, ignoring gender should be seen as a more suited strategy when men aim to obtain a wanted outcome on a task that is incongruent to their gender (e.g., women’s jewelry, Bear & Babcock, 2012), or when trying to avoid chores stereotypically assigned to men (e.g., molding the lawn, Demoulin & Teixeira, 2016). Future studies should put this theoretical model to a test and empirically explore how men’s gender approach might vary along with the task’s congruence to their gender and the expected outcomes in negotiation.

Relatedly, future research should shed new light on how men’s gender approach impacts women’s gender ideologies in negotiation. Indeed, literature reveals women negotiators to react in a more emotional manner than men (Stuhlmacher & Walters, 1999) and often show more interest in interpersonal relationships in negotiations (Kray & Gelfand, 2009). As such, one would assume that women negotiators would be particularly sensitive to the (often implicit) message male counterparts send on how gender should be handled in negotiations and that they should adjust their overall behavior in function of their counterpart’s feedback. More specifically, a positive feedback that either acknowledges women’s place at the bargaining table (sexawareness) or redirects attention to other, non-gender related traits (sexblindness) should at times prove beneficial and likely elicit a similar (positive) gender approach from women negotiators. Unfortunately, past work warns that this is seldom the case, and women tend to receive worse treatments from male counterparts, even when applying similar bargaining strategies as men (Kray et al., 2014). Even more, when trying to exhibit the manly behavior required to fit this stereotypical profile of a successful negotiator, women often encounter backlash (Bowles et al., 2007). This obviously creates a double bind for women in negotiations and likely accounts for their poorer negotiating performances. Better understanding how women and men’s ideologies interact in negotiation is definitely an intriguing topic on our future research agenda.

In a similar vein, more research is needed to shed light on possible individual determinants of one’s gender ideologies in negotiation. More specifically, negotiation research is often conducted from the implicit assumption that negotiators are well-aware of what motivates their behavior. Literature however warns that this is not always the case, and people often have “blind spots” related to information, particularly ethics (Bazerman & Chugh, 2005). As such, it would not surprise if one’s gender approach at the bargaining table would vary as a function of their stereotypes and gender beliefs, or of other individual traits known to negatively affect one’s attitudes toward people in gender-incongruent roles (e.g., ambivalent sexism, Glick & Fiske, 1996; Glick, Diebold, Bailey-Werner, & Zhu, 1997; Glick, Wilkerson, & Cuffe, 2015, or social dominance orientation, Pratto, Sidanius, Stallworth, & Malle, 1994). Better understanding how such characteristics might affect one’s gender approach should prove particularly important.

Last, but not least, future research should obviously acknowledge the importance of societal prescriptions on one’s gender approach in negotiations. Indeed, the perceived norms are of the utmost importance to people’s ideological endorsement (Bourguignon et al., 2015). These prescriptions are however known to vary along with specific cultures. For instance, whereas most Nordic/Western societies explicitly promote the idea that women and men are equal, and one should focus on individual traits (rather than gender) within mixed-gender interactions, this perspective is overtly contradicted by other cultures, with obvious consequences on how gender is handled (*Global Gender Gap Report 2020*). As such, what is true for one culture might not adequately extend to another. The overall support for sexblindness, for instance, might prove less prevalent within less gender equality-preoccupied countries than it is in highly equalitarian ones. Future studies should find cross-cultural research to represent a particularly fascinating field of research on gender ideologies.

To be sure, our article is but a first step in what should be an intriguing, rewarding way to uncover gender ideology’s untapped potential in clarifying gender effects at the bargaining table. To the best of our knowledge, no other research has explored gender ideologies in the field of negotiation, and we are not aware of any studies focused upon the link among gender ideology and other negotiating matters. Clearly, further research should benefit from a more nuanced perspective on this unexplored, yet potentially important variable.

## Footnotes

Following past work on gender in negotiation, we use the concept of *gender effects* across the manuscript to refer to the differences among male and female negotiators. We could also talk of *sex effects* because we approach the differences among women and men in the biological sense of this dichotomy and not the socially constructed concepts corresponding to masculinity and femininity.

We use the terms “sexblindness” and “sexawareness” to refer to the two main gender ideologies. We nevertheless note that Hahn et al. (2015) chose the terms of “gender blind” and “gender aware” for the two concepts, thus continuing the path proposed by Malicke (2013) to distract attention from the biological differences and redirect it towards cultural ones. These same concepts are also used in several other studies that we present throughout the article. Although we find this idea intriguing, we consider the terms used by Koenig and Richeson (2010) as better suited to describe the two main gender ideologies. We argue that the concept of gender ideology denotes the framework people use in addressing the innate differences between women and men, rather than their cultural representations. Being sex (gender) blind or sex (gender) aware points out to whether the one’s sex should or should not be considered in mixed-gender interactions, rather than to whether the social construct of gender should or should not play a part in such interactions. We will therefore refer to the two ideologies as “sexblindness” and “sexawareness” throughout the paper.
